# High-resolution probe design for measuring the dielectric properties of human tissues

**DOI:** 10.1186/s12938-021-00924-1

**Published:** 2021-08-28

**Authors:** Xinran Wang, Hongfu Guo, Chen Zhou, Junkai Bai

**Affiliations:** School of Physics and Optoelectronic Engineering, Xidian University, Xi’an, China

**Keywords:** Near-field probe, Spiral resonator, Dielectric properties, Body measurement, Miniaturization

## Abstract

**Background:**

In order to use the microwave to measure the dielectric constant of the human body and improve the measurement resolution, a small near-field probe working at 915 MHz is designed in this paper.

**Method:**

Based on the electric small loop antenna model loaded by the spiral resonator (SR), a small near-field probe was designed. The probe model is designed and optimized by the HFSS (high frequency structure simulator) software. The human tissues were tested by the manufactured probe and the relationship between the S11 parameters of the probe and the human tissues was analyzed.

**Results and conclusions:**

A probe with small size was designed and fabricated, with the overall size of 10.0 mm × 12.0 mm × 0.8 mm. The probe has a good performance with a 30.7 dB return loss, a 20 MHz bandwidth at the resonance point, and a distance resolution of 10 mm. Due to the small size and good resolution of the probe, it can be used in the measurement of human tissues.

## Background

The human body is composed of a variety of biological tissues, such as skin, fat, muscle, etc. The dielectric properties are inherent property of biological tissues. Physiological changes of the water content, protein content, types and cell structure of biological tissues will lead to changes in its dielectric properties. A large number of studies have shown that the dielectric properties of different types of biological tissues are different, and the dielectric properties between normal tissues and diseased tissues are also quite different [1, 2].

As a new type of physiological signal detection method, microwave has a broad application prospect in the fields of medicine and biology. Researches on dielectric property of tissues is the key to the development of an accurate microwave-based anomaly detection systems [3]. The open-ended coaxial reflectometry method [4, 5] is widely used in the study of the dielectric properties of biological tissues. Therefore, the dielectric properties are measured in this paper.

In general, we cannot directly measure the dielectric properties of human body, but we can use the probes to measure other parameters of the human body to obtain the dielectric properties indirectly [6]. S11 parameter is one of the important parameters of the antenna, which is the reflection caused by the change in dielectric constant. Its value is equal to the ratio of the reflected wave power and the incident wave power at the transmission line port. In the measurement of human tissues, the probe is placed on the surface of the human body. According to the wave propagation theory, the electromagnetic wave radiated by the probe is incident on the human tissue to be measured. Tissues with different dielectric properties absorb different electromagnetic waves and reflect different electromagnetic waves, resulting in different S11 parameters. Therefore, the corresponding tissue dielectric properties can be analyzed by measuring the changes of S11 parameter [7].

At present, there are three main types of dielectric constant measurement methods commonly used: resonance method, short-circuit waveguide method and reflection method. Microwave-resonance method [8] and short-circuit waveguide method [9] have strict requirements on the size of the measured object, and the measured tissue needs to be cut into a shape fit for measurement. Therefore, these methods are difficult to deal with the measurement of human tissues. Among the reflection methods, the most commonly used method is the open-end coaxial method [10]: Abdilla et al. used the open-ended coaxial method to measure the dielectric properties of muscle and liver tissue from 500 MHz to 40 GHz [11]. Fornes-Leal et al. measured the dielectric properties of malignant colonic tissue using an open-ended coaxial probe of 0.5–18 GHz [12]. These measurement results provide references for the design and research of subsequent probe. The open-ended coaxial method needs to select an appropriate calibrated nominal when it is applied, and its calculation accuracy has a low tolerance for errors. When measuring human tissue with the open-ended coaxial method, the measured surface needs to meet the condition of approximately infinity, which is a rigorous condition. In addition, there are measurement methods such as horn probe [13] and microstrip array antenna [14]. However, compared with the abnormal human tissues, they usually have large size, low resolution, and poor detection.

**Fig. 1:**
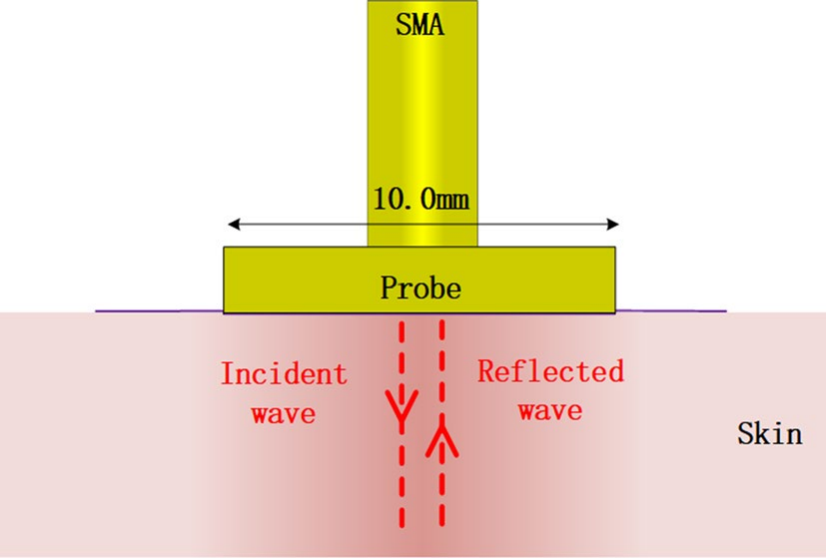
Schematic diagram of measuring human tissues by reflection method

The probe design in this paper is based on the reflection method, which is combined with a resonator [15]. Figure 1 shows the schematic diagram of measuring human tissues by reflection method. In order to reduce the size of the probe and improve the resolution of the probe, a waveguide with resonant characteristics can be loaded on the probe antenna, such as folded metal strips [16, 17] and planar microwave resonators [18–20]. At present, some researches on planar resonators have been applied to practical application scenarios, such as near-field induction based on planar resonators, material characterization, filter design, etc. These resonators include split-ring resonators (SSR) [21], complementary split-ring resonators (CSSR) [19], spiral resonators (SR) [22] and electric LC resonators [23, 24]. The design of these resonators ensures the miniaturization of microwave probes because the size of the resonators is very small compared to the wavelength at operating frequency.

In this paper, a high-resolution probe operating at 915 MHz is designed and fabricated, which is based on an electric small loop antenna loaded with a circular spiral resonator. The probe has the following novelties and development prospects: (1) probe’s working frequency can be adjusted by modifying the resonator coil parameters and matching capacitor parameters. Unable elements such as varactor diodes can also be loaded on the structure to make a frequency adjustable probe. (2) In the measurement, the probe can be located at a certain distance above the skin surface, which can avoid direct contact with the human body and the influence of pressure on the measurement results. (3) The probe adopts a planar circuit structure, which is easy to integrate with other planar circuits (such as microstrip lines and coplanar waveguides), and has great application prospects. The human tissues were measured by the designed probe. The HFSS simulation model of the probe is given, and the mediators simulation is carried out. The consistency between the simulated and measured results proves that the designed probe can be applied to the measurement of human tissues.

## Results

### Probe creation and testing

**Fig. 2:**
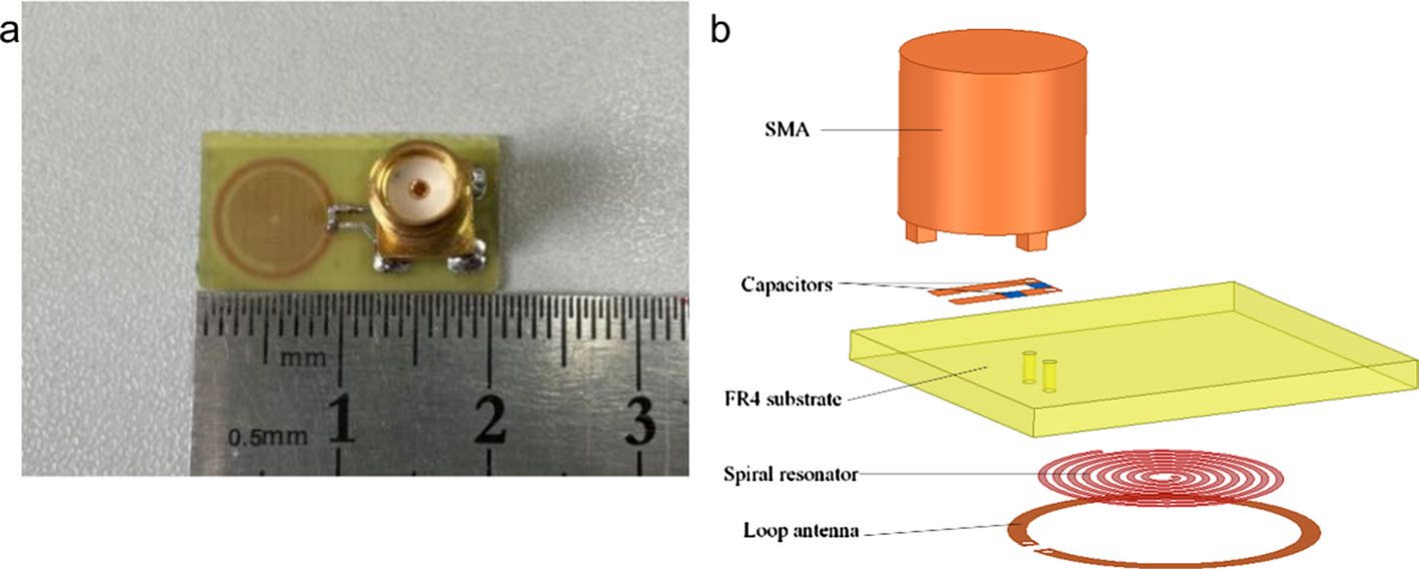
**a** Fabricated probe prototype; **b** exploded schematic of the probe

The fabricated probe is shown in Fig. 2. The probe consists of a 7-turn spiral resonator (7-SR) with a width of 0.2 mm and a small loop antenna with a width of 0.5 mm which is designed around the SR to excite it. The SR and loop antenna are made of copper strips laid on a 0.8-mm-thick FR4 substrate. The opening of the loop antenna is composed of two 0402 patch capacitors (*C*_1_ and *C*_2_) to match the probe to the feeding 50-Ω line. The capacitance values of *C*_1_ and *C*_2_ are 10 pF and 25 pF, respectively. Unlike most resonators used for near-field imaging in the past, the probe designed in this article uses only one port to power supply.

**Fig. 3:**
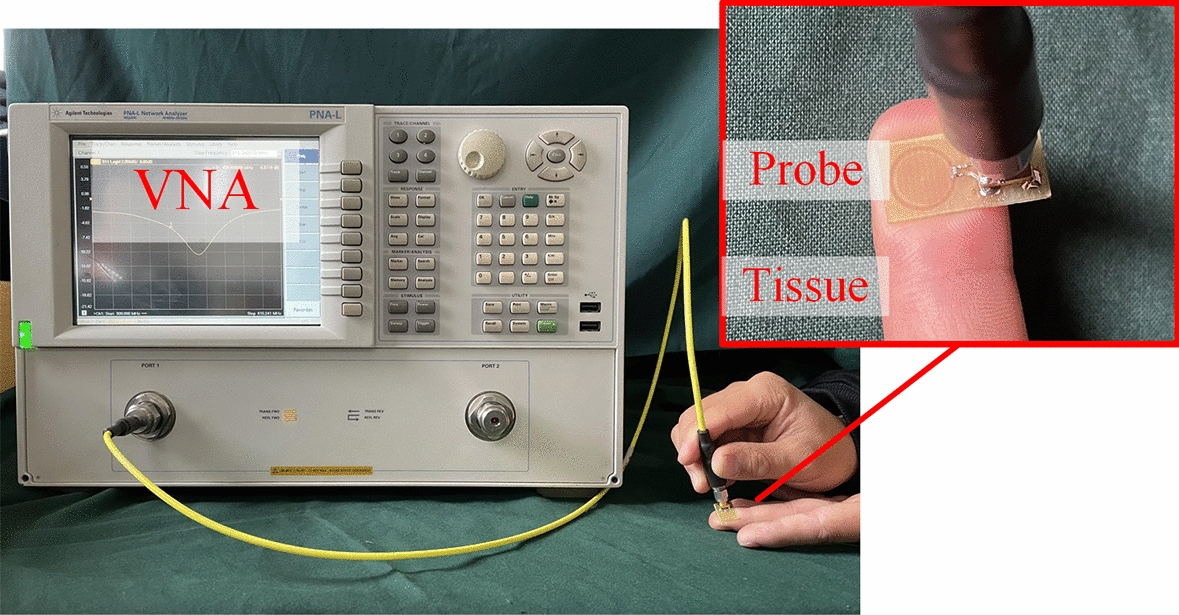
Experiment system for measuring tissues

The experimental system for measuring human tissues is shown in Fig. 3. The probe is connected to the N5230C VNA (vector network analyzer), and the microwave signal is fed into the matching circuit and antenna through the SMA connector. The tissue to be measured is placed under the probe, and it can be analyzed through the S11 parameter curve obtained on the VNA. During the measurement process, the interference objects are kept away from the probe to minimize the impact of the environment on the probe.

**Fig. 4:**
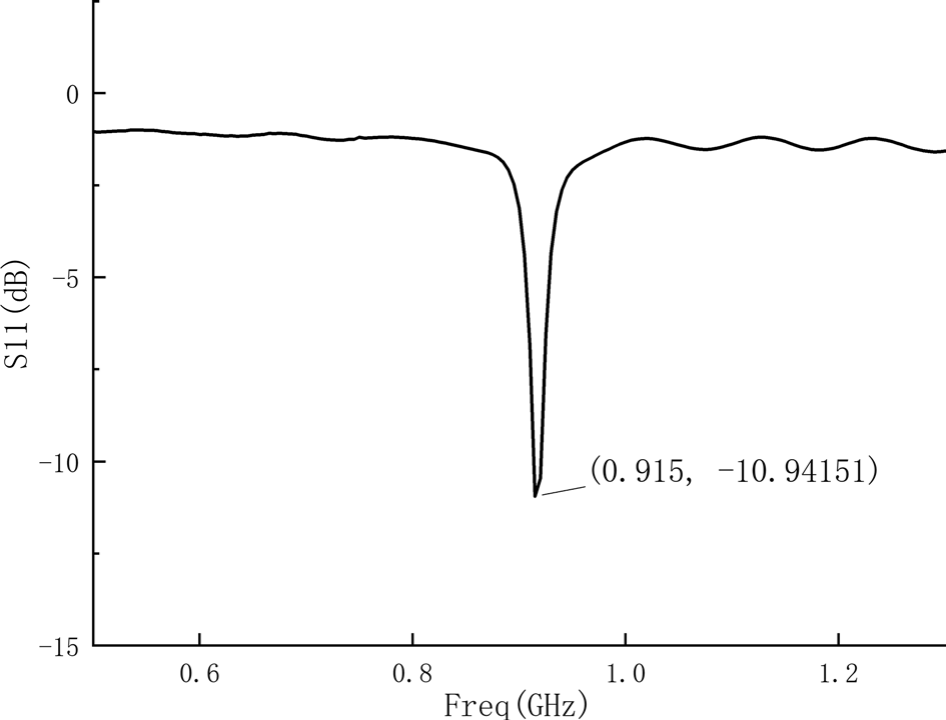
The measured S11 parameters of the probe

When the probe is connected to the VNA without placing any objects under it, the S11 parameter of the probe can be obtained on the VNA as shown in Fig. 4. It can be seen from the figure that the resonant frequency of the probe antenna is 915 MHz, and the S11 parameter of the resonance point is − 10.9 dB. The probe can be used for the measurement of human tissues, and has been miniaturized with a resolution of 10 mm.

### Application of probe in measuring tissues

The tissues were measured by the measurement system shown in Fig. 3. In order to prevent the probe from directly contacting the measured tissue, an insulating wave-transmitting material with a thickness of 0.05 mm was pasted on the surface of the probe.

#### A. Application of probe in measurement of different biological tissues

**Fig. 5:**
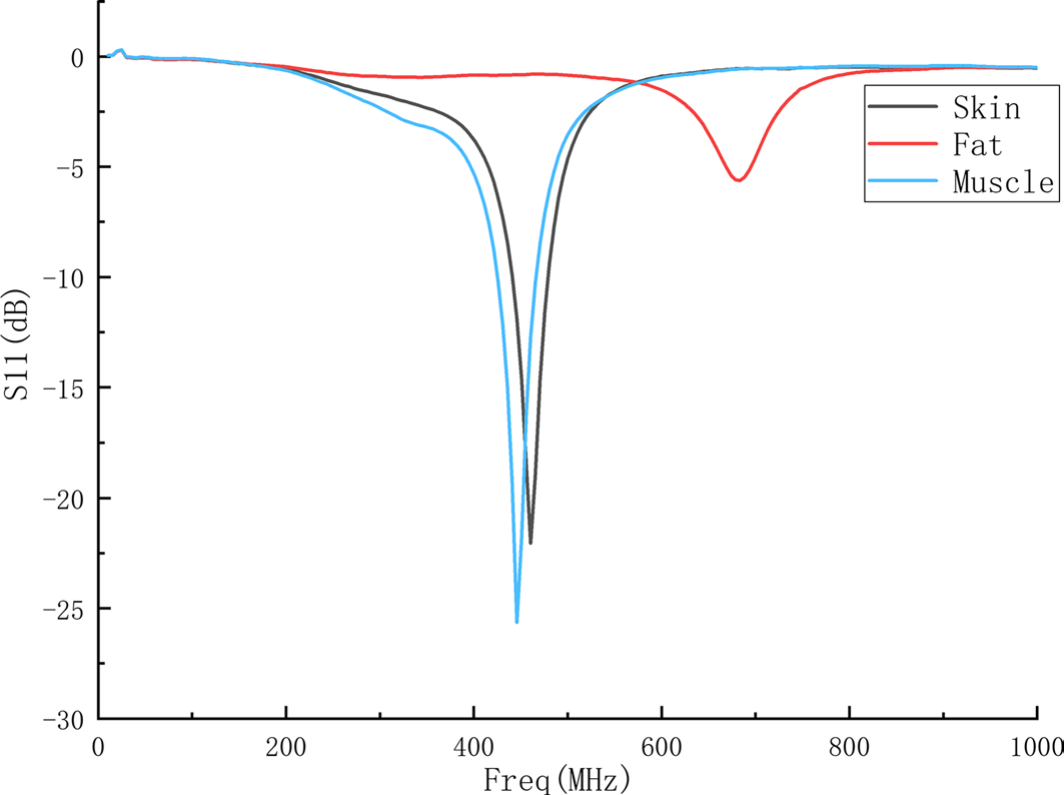
S11 parameters of different tissues

It is difficult to directly measure each tissue layer of human body by probe. In order to verify that the probe can be used to measure the dielectric constant change of human tissue, pork was selected as the measurement object, which is similar to human tissue structure. The skin, fat and muscle of pork were measured, respectively, with the probe, and the S11 parameters were obtained as shown in Fig. 5.

In order to obtain the relationship between the probe S11 parameter and the dielectric constant of the tissue more intuitively, the resonant frequencies of the probe in each tissue and the dielectric constants of the probe in each resonant frequency (reference value rather than exact value) are listed in Table 1; [25].

**Table 1:** Parameters of different tissues

Tissue	Fat	Skin	Muscle
Dielectric constant (reference value)	≈ 5	≈ 45	≈ 55
Resonant frequency (MHz)	683.2	460.5	445.6

It can be seen from the table that the resonant frequency of the probe decreases with the increase of the dielectric constant of the biological tissue, which verifies that the designed probe can be applied to the measurement of different biological tissues.

#### B. Application of probe in measuring human tissue with different thickness

**Fig. 6:**
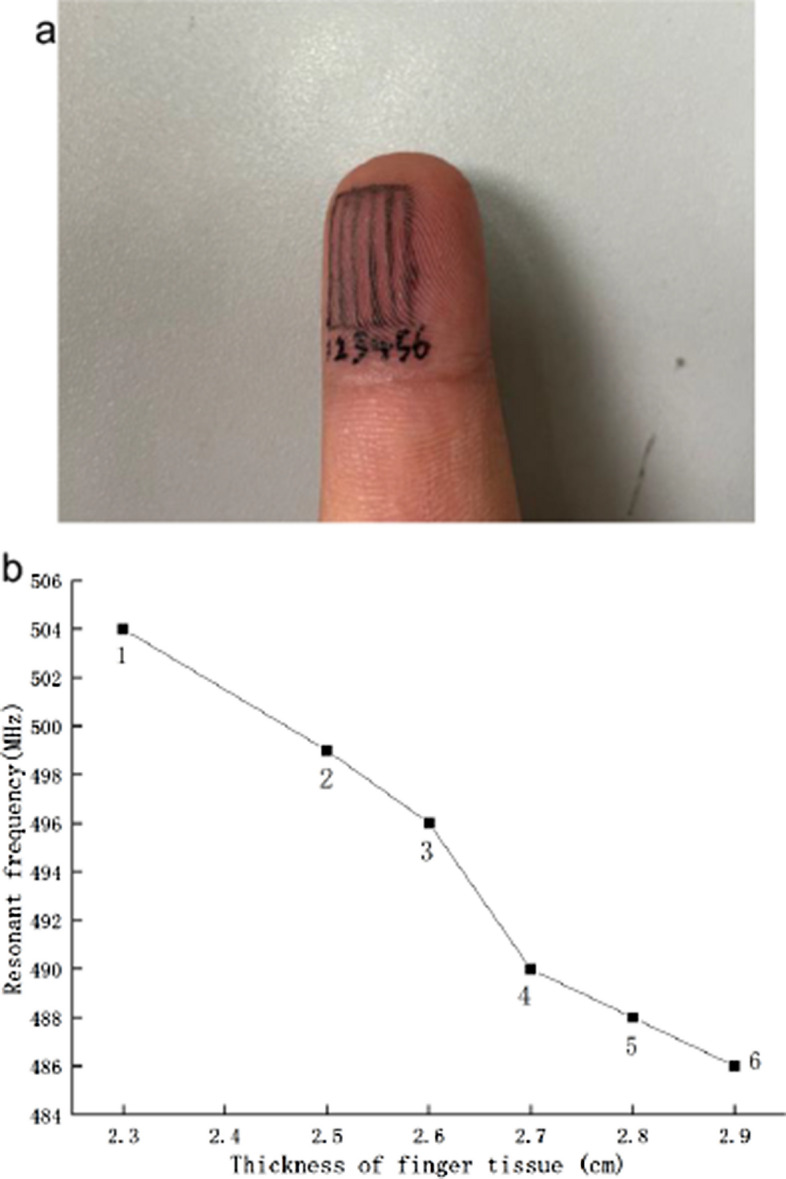
**a** Photo of finger with six measurement points marked; **b** relationship curve between resonance frequency and finger thickness

In order to verify that the probe can be used to measure the thickness change of human tissues and to verify the resolution of the probe, the finger pulp of the human body is selected as the measurement object. Take six points from the edge of the finger pulp to the center of the finger pulp at 1 mm intervals (Fig. 6a).

An ultrasound probe was used to measure the thickness of the tissue at each point of the finger pulp, and the fabricated probe was used to measure the S11 parameter at each point. In order to obtain more accurate results, three repeated experiments were carried out, and the maximum resonant frequency of each measuring point was selected. The relationship between the resonant frequency of the probe and the thickness of the finger tissue was obtained as shown in Fig. 6b.

It can be seen from the figure that the resonant frequency of the probe decreases as the thickness of the finger pulp increases, which verifies that the designed probe can be applied to measure the thickness of human tissues.

#### C. Application of probe in nodule measurement

**Fig. 7:**
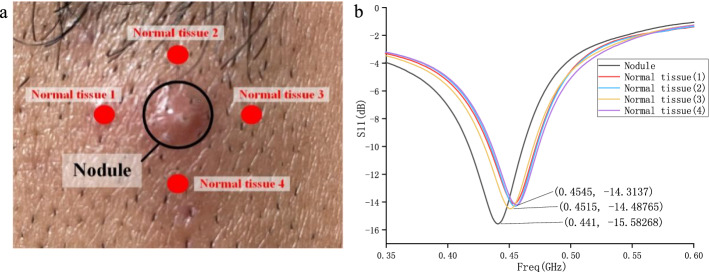
**a** The measured nodule tissue; **b** comparison of S11 parameters between normal tissues and nodules

In order to verify that the probe can be used to measure abnormal nodules in human tissues, a nodule on human skin is selected as the measurement object (Fig. 7a). The nodule is located on the edge of the subject’s face and is about 4 mm in diameter. The measured S11 parameters are shown in Fig. 7b. It can be seen from the figure that the difference in the resonance frequency obtained by the probe measuring the normal tissue of the same part of the human body is small, the difference is about  ±  3 MHz. When measuring abnormal nodules, the resonance frequency is reduced by more than 10 MHz compared with the normal tissue. Therefore, the existence of abnormal nodules can be determined by the range of probe resonance frequency offset.

## Discussion

### Model of probe applied to the measurement of tissues

**Fig. 8:**
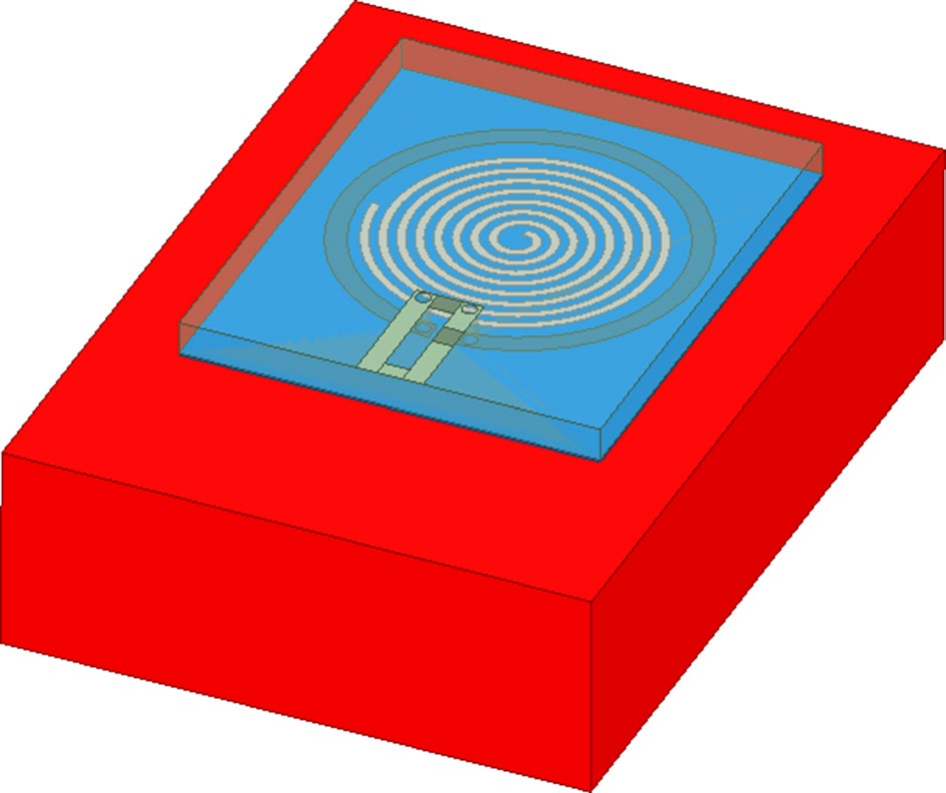
Simulation model of human tissue measurement

In order to verify the consistency between the experimental results and the theory, a probe for measuring human tissue model is established in HFSS (Fig. 8). The probe model was placed above the human tissue layer. In order to prevent the probe from directly contacting with the human tissue layer, a 0.05-mm insulating film is added between the probe and the human body, and its size was consistent with that of the probe. From top to bottom, the simulation model is the probe, the insulating film and the human tissue layer.

### Simulation analysis of probe applied to the measurement of tissues

#### A. Simulation of probe measuring different biological tissues

**Fig. 9:**
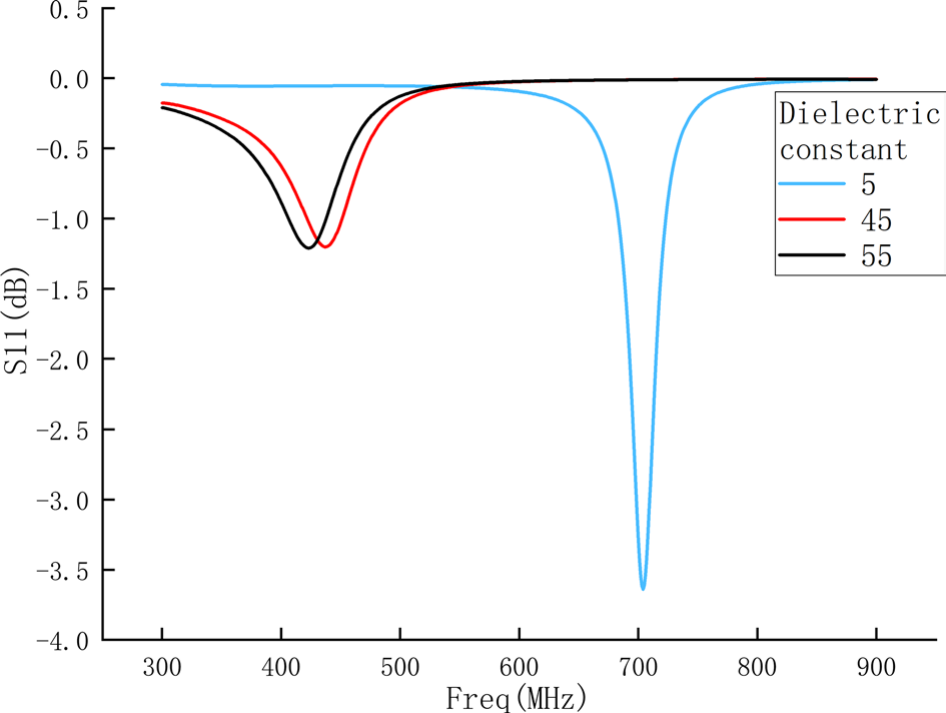
S11 parameters of human tissues with different dielectric constants

In order to obtain the theoretical results of measuring different biological tissues by the probe and compare with the experimental results, the relative permittivity of the human tissue layer was set to 5, 45 and 55 for simulation. The S11 parameters are shown in Fig. 9.

It can be seen from the figure that the resonant frequency of the probe decreases as the dielectric constant of the human tissue layer increases, which is consistent with the measurement results in Fig. 5. The difference between the S11 parameter value in the measured results and the simulated results is because the dielectric constant used in the simulation is the reference value instead of the real value of the measured pork, and the follow-up research will carry out further calibration.

#### B. Simulation of probe measuring human tissue with different thickness

**Fig. 10:**
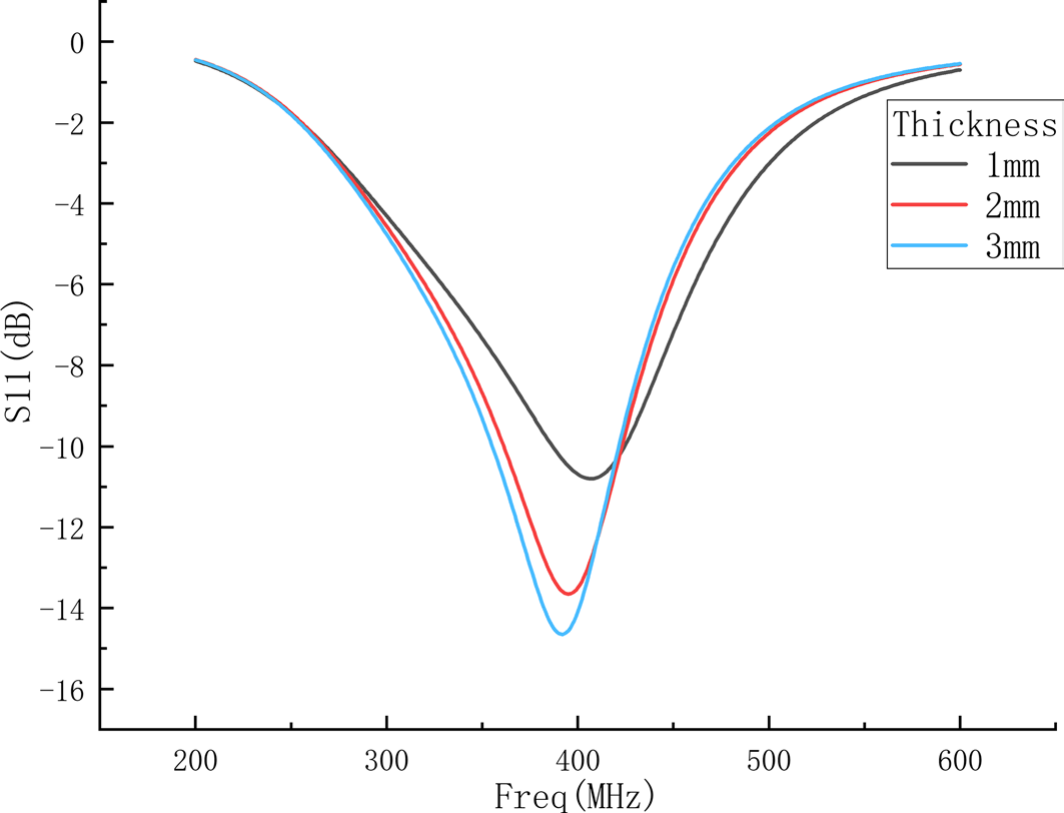
S11 parameters of human tissues with different thicknesses

In order to obtain the theoretical results of measuring different thicknesses of human tissues by the probe and compare with the experimental results, the dielectric constant of the human tissue layer is set to 40 and remains unchanged, and the thickness is set to 1 mm, 2 mm and 3 mm for simulation. The thickness increased from 1 to 5 mm with 1 mm intervals. The S11 parameters are shown in Fig. 10.

It can be seen from the figure that the resonant frequency of the probe decreases as the thickness of human tissue increases, which is consistent with the measurement results in Fig. 6b. The value of S11 will be further calibrated in subsequent research.

#### C. Simulation of probe measuring nodule

**Fig. 11:**
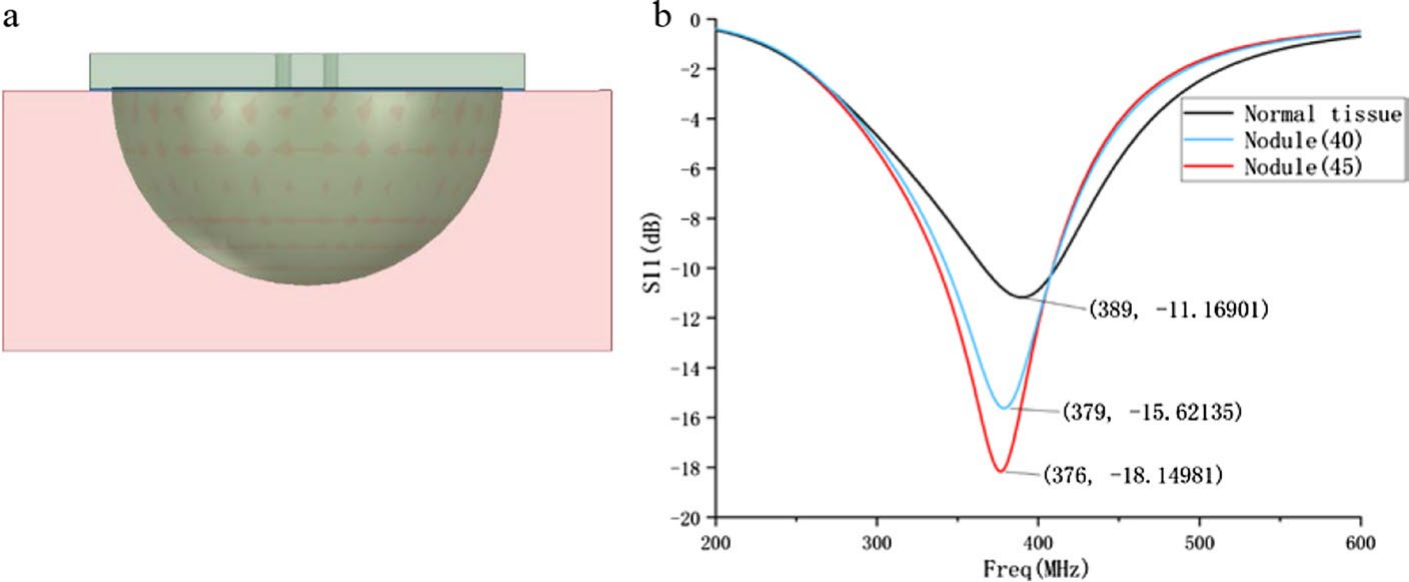
**a** Simulation model of measuring nodule; **b** comparison of S11 parameters of human tissue layers with and without nodules

In order to obtain the theoretical results of the probe measuring human nodules and compare them with the experimental results, a hemispherical nodule with a diameter of 9 mm is established in the human tissue layer (Fig. 11a). The dielectric constant of the human tissue layer is set to 30. According to the literature [6], the dielectric constant of abnormal tissues is 30–50% larger than that of normal tissues. Therefore, the dielectric constants of hemispherical nodules are set to 40 and 45, respectively. The S11 parameters obtained after the simulation are shown in Fig. 11b.

It can be seen from the figure that when the human body has abnormal nodules with a dielectric constant 30–50% larger than normal tissues, the resonance frequency of the probe is reduced by 10–13 MHz, which is consistent with the measurement results in Fig. 7b. Therefore, the existence of abnormal nodules can be determined by the range of probe resonance frequency offset.

According to the experimental and simulation results, when the dielectric properties and thickness of the measured biological tissues change, the S11 parameters of the probe have regular changes; when nodule appears on the surface of the human body, the resonance frequency of the probe changes significantly. The consistency of the simulation and experimental measurement results proves that the designed probe has practical application value. The error is due to the fact that the dielectric constant of the tissue in this paper is a reference value rather than an accurate value. The probe will be calibrated subsequently. After calibration, the dielectric constant will be inversely calculated and the human tissue can be accurately measured.

At present, there have been some studies on the electrical characteristics of acupoints [26]. Based on these studies, we speculate that the dielectric characteristics of human acupoints may change. In order to enable the probe designed in this paper to be used for the measurement of acupoints, we will conduct more in-depth research on the dielectric properties of human acupoints in the future. At the same time, in order to enable the probe to accurately measure human tissues, in future research we will consider combining numerical calculations to further optimize the probe numerically.

## Conclusions

A near-field probe antenna loaded with a circular SR is designed. The resonant frequency of the probe is 915 MHz. In order to verify that the probe can be used to measure the dielectric properties of human tissues, the fabricated probe is used to measure pork, human fingers and special nodules. When the dielectric properties and thickness of the measured object change, the S11 parameters of the antenna have obvious regular changes. The probe model is established by HFSS, and the influence of the change of the dielectric properties of human tissues on its S11 parameters is also simulated and analyzed. The consistency between the simulation and the experimental measurement results proves that the designed probe has practical application value.

Due to the small size and good resolution of the probe, it can be used in the measurement of human tissues with different thicknesses and abnormal nodules, and can also be used in the diagnosis of traditional Chinese medicine acupoints. It is worth noting that the designed probe is suitable for flat and relatively uniform human tissues, such as limbs and abdomen. It is not suitable for parts with complex structures or obvious protrusions, such as joints. A flexible and stretchable antenna for bio-integrated electronics [27] has been recently developed, which uses new elastic materials to replace traditional rigid materials. Additionally, there are some rapid fabrication of these antennas such as laser micromachining technology [28] and laser-induced graphene [29]. In order to solve the limitations of the probe designed in this article, we will consider using these advanced materials and technologies to optimize the probe in the follow-up, so that the probe can be used in complex parts of the human body.

## Methods

### Establishment of the model

The probe design in this paper is based on the reflective sensor, which is combined with a resonator. The SR is used as the resonant structure of the probe because it could provide a higher miniaturization rate compared to other resonant structures. For example, with the same number of turns and side length, the SR would resonate at lower frequency compared to the SRR [30]. In addition, the SR can be excited by a single port, which reduces the complexity and cost of the system. We use a small loop antenna placed around the SR to excite the SR. The loop antenna generates a time-varying magnetic field perpendicular to the loop plane, which excites an induced current in the spiral resonator [31].

**Fig. 12:**
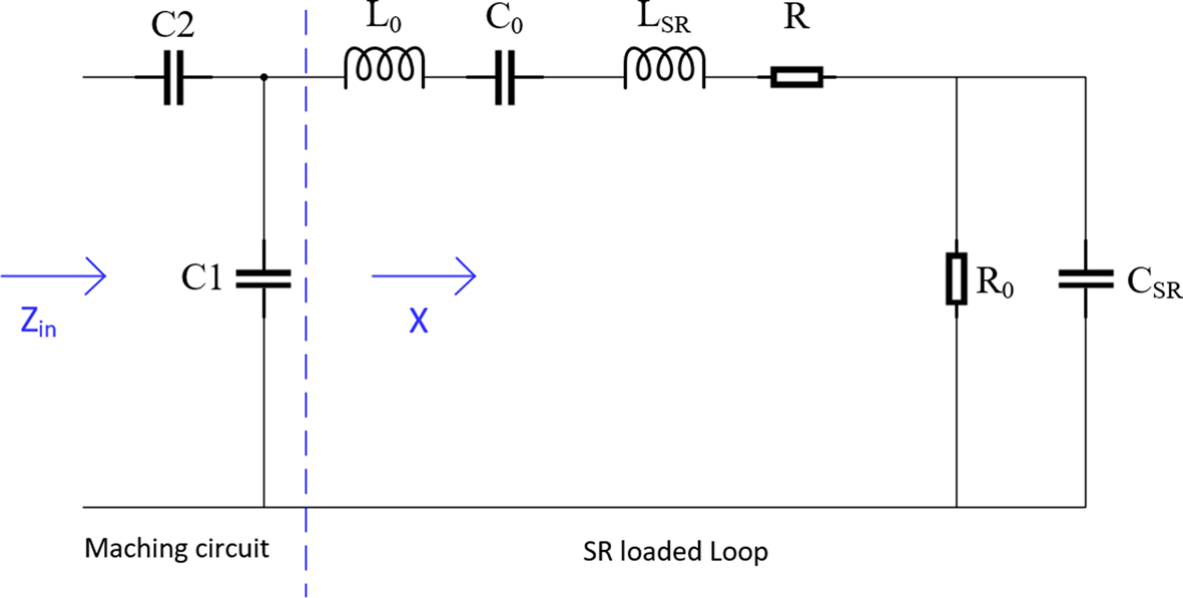
Equivalent circuit model

According to the principle of loop antenna and spiral resonator theory, the simulation model of loop resonant antenna is established, which reduces the probe size and improves the spatial resolution. The equivalent circuit of the model is shown in Fig. 12, and the equivalent impedance is given by

$$ Z_{{{\text{in}}}} = \frac{{1 + \omega C_{1} X + \omega C_{2} X}}{{\omega C_{2} \left( {1 + \omega C_{1} X} \right)}}, $$

$$ X = \frac{{R_{0} }}{{1 + \omega C_{{{\text{SR}}}} }} + \frac{1}{{\omega C_{0} }} + \omega \left( {L_{0} + L_{{{\text{SR}}}} } \right) + R, $$

where *Z*_in_ is the equivalent impedance of the model, *X* is the equivalent impedance of the SR loaded loop, *C*_0_ represents the stray capacitance between the loop and the *SR*, *L*_0_ represents the inductance of loop, *L*_SR_ represents the inductance of the SR, *R* represents the ohmic losses in the loop and SR conductors, the dielectric loss and stray capacitance between the SR loop turns are modeled by the lumped elements *R*_0_ and *C*_SR_, respectively. By changing the parameters of *C*_1_ and *C*_2_, the probe can obtain impedance matching at the best operating frequency.

**Fig. 13:**
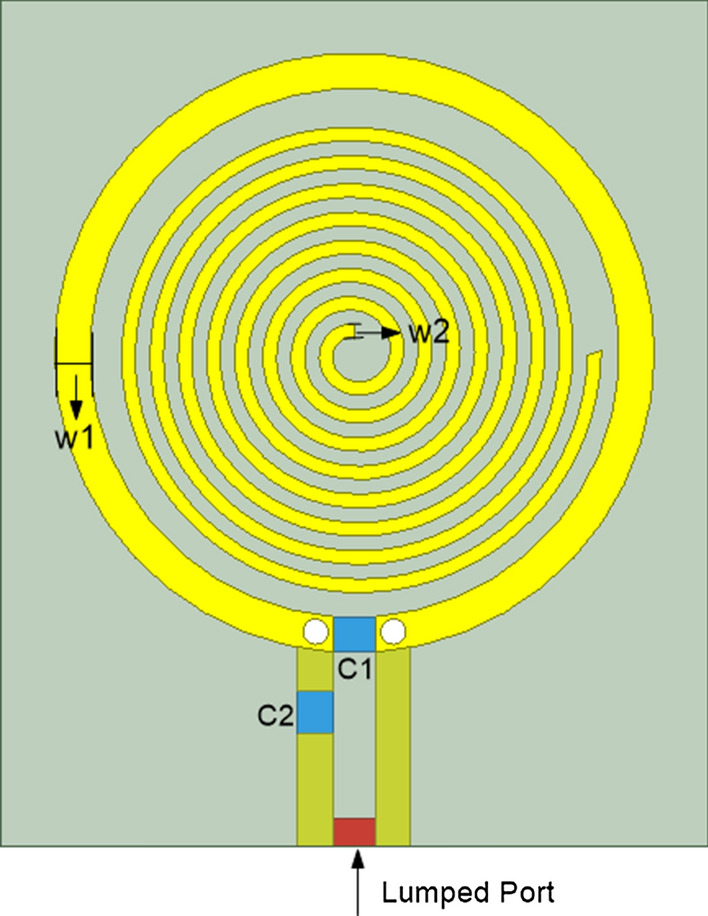
Simulation model of near-field probe

Based on this, a simulation model is established in HFSS, as shown in Fig. 13. The designed antenna model of human tissue dielectric probe is composed of a circular SR and an annular antenna around the resonator. The antenna is laid on a 0.8-mm-thick FR4 substrate, and a 50-Ω lumped port excitation is added at the opening. The matching circuit is composed of two capacitors (*C*_1_ and *C*_2_) at the opening of the loop antenna.

In antenna design, S11 parameters are usually used to reflect the matching characteristics of the antenna. The relationship between S11 and input impedance is:

$$ {\text{S}}11 = 20{\text{log}}\frac{{Z_{{{\text{in}}}} - Z_{0} }}{{Z_{{{\text{in}}}} + Z_{0} }}, $$

where $Z_{0}$ is the characteristic impedance of the transmission line (50 Ω). It can be seen that when the input impedance of the probe is closer to 50 Ω, the smaller the value of S11 is, the closer the probe is to the matching state.

In this paper, S11 is simulated by HFSS. The specific size of the probe and the matching capacitance value are adjusted to make the value of the S11 parameter at the resonance point as small as possible, and finally adjust the probe to the best matching state.

### Selection of design parameters

According to FCC regulations on the ISM band, 433 MHz, 915 MHz and 2450 MHz can be used in the medical applications. According to the relationship between frequency and wavelength and the relationship between wavelength and penetration depth, electromagnetic waves of different frequencies penetrate human tissues at different depths. According to the principle of microwave propagation, the higher the frequency, the weaker the penetration of the probe. The probe designed in this paper is used for abnormal measurement of subcutaneous tissues and acupoints. Therefore, the working frequency of the probe is set to 915 MHz.

According to the application requirements of traditional Chinese medicine (TCM) acupoint detection, the resolution of the probe designed in this paper should not be greater than 10 mm, so the width of the substrate of the probe is set to 10 mm. The diameter of the loop antenna should be smaller than the width of the substrate, so the diameter of the loop antenna in the probe design is set to 8 mm. In order to facilitate the welding of the 0402 capacitance in the matching circuit, the width of the loop antenna w1 is set to 0.5 mm.

#### A. Optimization of the SR

After determining the outer dimensions of the probe and the size of the loop antenna, the parameters of the SR are discussed. The resonant frequency of the SR is given by

$$ f_{0} = \frac{1}{{2\pi \sqrt {LC} }}, $$

where *L* and *C* are the effective inductance and capacitance of SR. Some of the most important parameters that can affect the resonant frequency of the SR are the width and the number of turns [32]. These parameters of the SR are optimized using the optimization tool of HFSS.

**Fig. 14:**
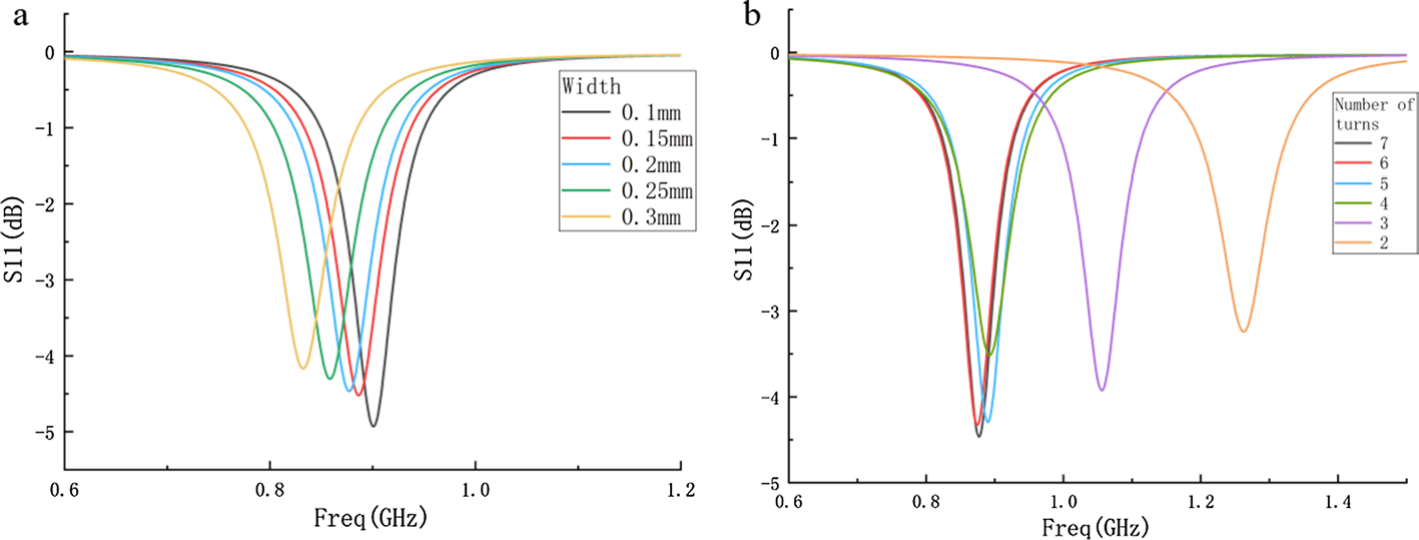
**a** S11 parameters of probe with different width of resonator; **b** S11 parameters of probe with different turns of resonator

The width w^2^ of the SR is increased from 0.1 mm to 0.3 mm at an interval of 0.05 mm. The variation of the S11 parameter of the antenna is shown in Fig. 14a. It can be seen from the figure that the smaller the width of the SR, the smaller the S11 value of the resonant point. The width of the resonator remains unchanged, and the number of cycles is increased from two to seven. The variation of S11 parameters of the antenna is shown in Fig. 14b. It can be seen from the figure that the S11 parameter of the resonant point is the smallest when the number of turns of the SR is seven.

Considering that the minimum width of the copper wire in actual production is about 8 mil, the width of the SR is selected as 0.2 mm. The number of turns of the SR is selected as seven circles.

#### B. Optimization of resonance frequency

**Fig. 15:**
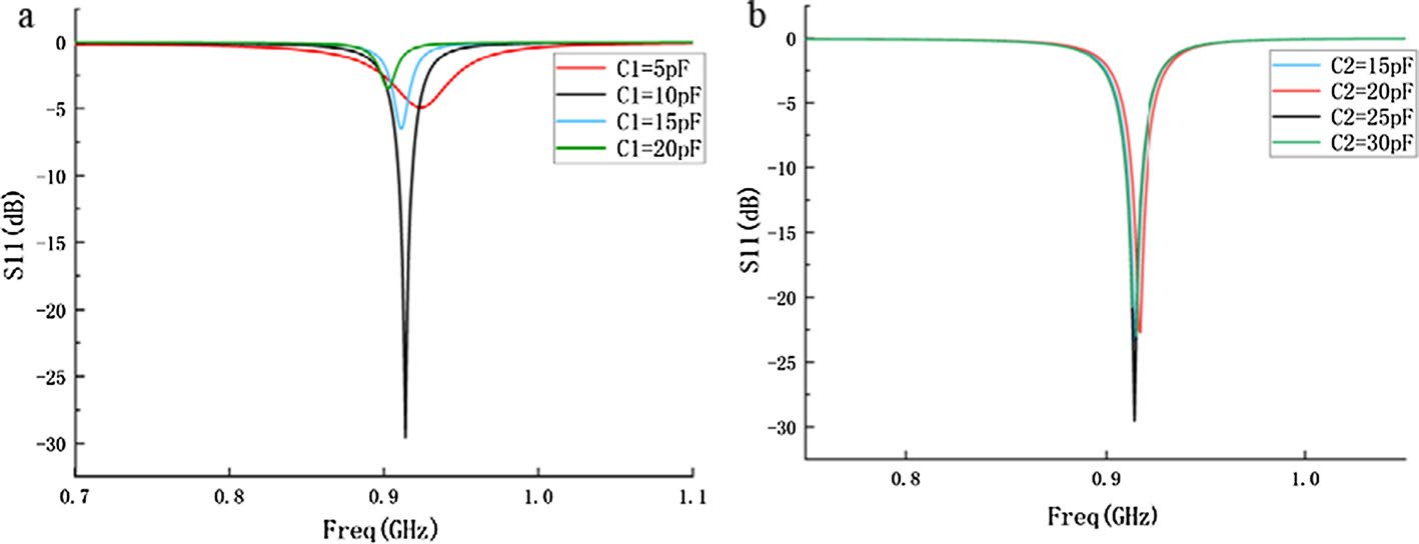
**a** S11 parameters of probe when *C*_2_ value changes (*C*_1_  =  10pF); **b** S11 parameters of probe when *C*_1_ value changes (*C*_2_  =  25pF)

In order to make the resonant frequency of the probe reach the required 915 MHz, a matching circuit is added to the probe to optimize the resonant frequency of the probe. Through simulation optimization (Fig. 15), when *C*_1_  =  10 pF, *C*_2_  =  25 pF, the resonant point of the probe is the most ideal, and the resonant frequency is closest to 915 MHz. The resonant frequency is 914 MHz with the − 10 dB bandwidth of 10 MHz which covers the required 915 MHz operating frequency.

**Fig. 16:**
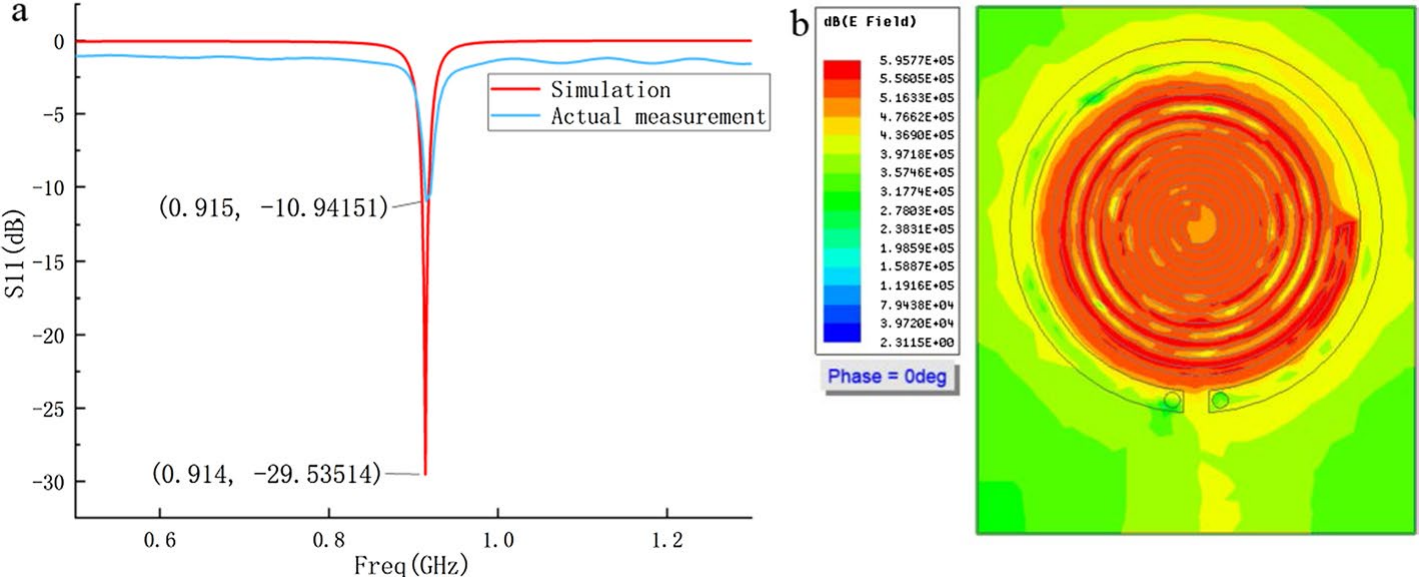
**a** Comparison of S11 parameters between simulation and measurement; **b** simulated electric field distribution on the probe surface

The comparison of S11 parameters between simulation and measurement is shown in Fig. 16a. Through conversion, when S11 is less than − 10 dB, the ratio of the reflected wave power of the port to the incident wave power is less than 0.1. In antenna design, it is usually specified that S11 is less than − 10 dB to meet the requirements. The S11 values of the probe obtained by experiment and simulation are less than − 10 dB at the resonant point, so the probe meets the design requirements. These deviations are caused by the error between the actual welding capacitance and the simulation capacitance, and the error between the parameters of the actual plate-making material and the parameters of the dielectric substrate in the simulation. The simulated electric field distribution on the probe surface is shown in Fig. 16b. It can be observed that the electric field is more concentrated near the SR structure.

## Data Availability

Not applicable.

## Abbreviations

SR: Spiral resonators
SSR: Split-ring resonators
CSSR: Complementary split-ring resonators
ISM: Industrial Scientific Medical
TCM: Traditional Chinese medicine
VNA: Vector network analyzer
FCC: Federal Communications Commission
